# Supplementary material for: Anti-Atherogenic Actions of the Lab4b Consortium of Probiotics In Vitro
Source: Int J Mol Sci. 2023 Feb 11;24(4):3639. doi: 10.3390/ijms24043639 (PMC9964490; doi:10.3390/ijms24043639)
Supplement: Supplementary file 1 [file ijms-24-03639-s001.zip › ijms-2045687-supplementary.pdf]

Supplementary Data

Anti-atherogenic actions of the Lab4b consortium of probiotics *in vitro*

Victoria L. O'Morain [1], Jing Chen [1], Sue F. Plummer [2], Daryn R. Michael [2] and Dipak P. Ramji [1,*]

[1] Cardiff School of Biosciences, Cardiff University, Sir Martin Evans Building, Museum Avenue, Cardiff CF10 3AX, UK

[2] Cultech Limited, Unit 2 Christchurch Road, Baglan Industrial Park, Port Talbot SA12 7BZ, UK

* Correspondence: Professor Dipak P. Ramji, Cardiff School of Biosciences, Cardiff University, Sir Martin Evans Building, Museum Avenue, Cardiff CF10 3AX, UK. Tel: 0044 (0)29 20876753; Email: Ramji@Cardiff.ac.uk

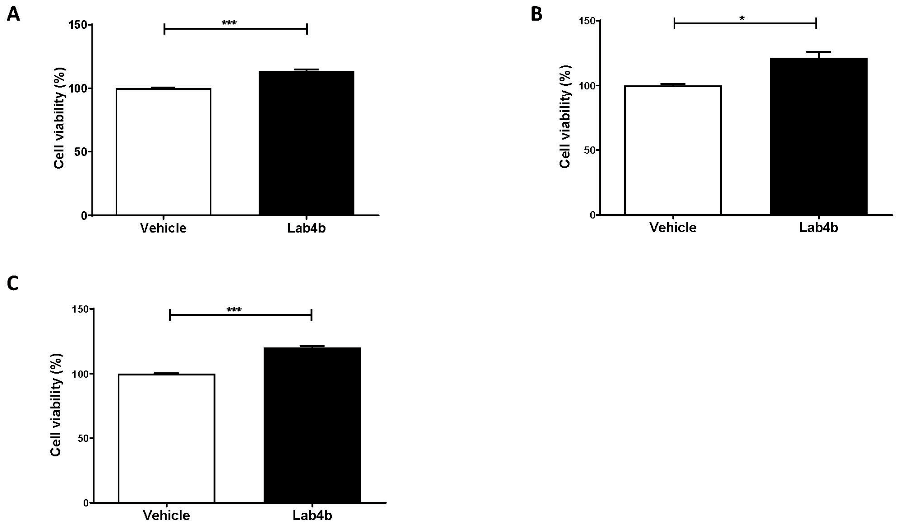

Figure S1. Lab4 CM had no detrimental effect on viability of all cell culture model systems used. THP-1 macrophages (A), HMDM (B), and HASMC (C) were treated for 24 h with vehicle or 1.5 µg/mL of Lab4b CM. Cell viability was determined by following the release of the LDH enzyme into the medium. Cell viability is shown as a percentage relative to the vehicle control, which was arbitrarily assigned as 100%. Data are mean ± SEM from three independent experiments, and statistical analysis was carried out using an unpaired Student's t-test (A,C) or Mann–Whitney test (B) (*, $p \leq 0.05$; ***, $p \leq 0.001$).

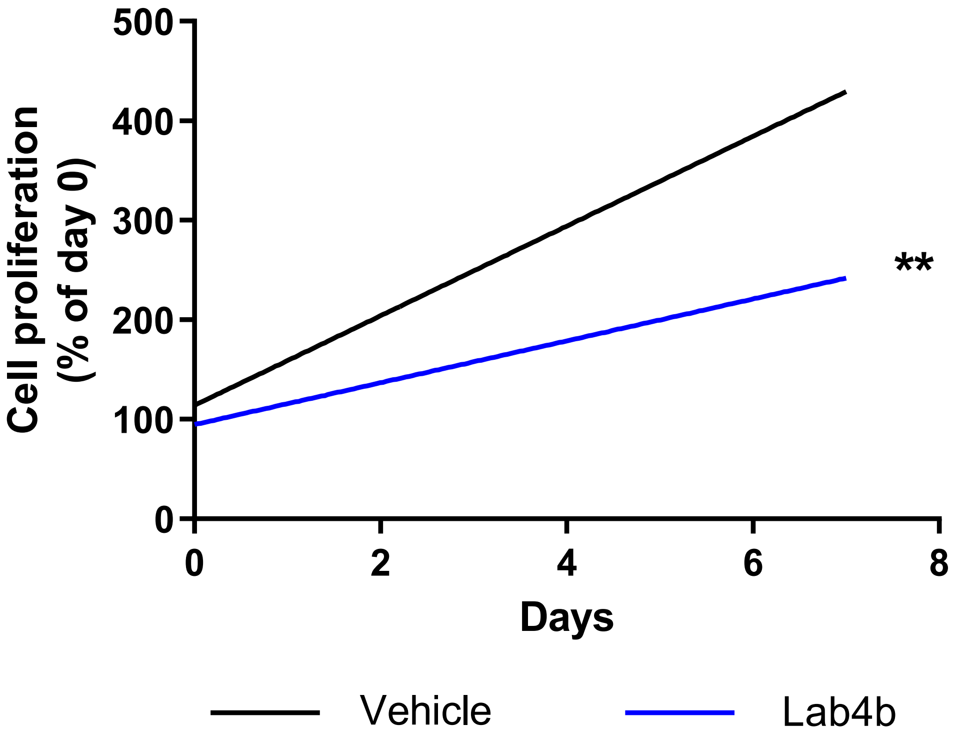

Figure S2. Lab4b CM decreased the proliferation of THP-1 monocytes over a 7-day period. THP-1 monocytes treated with either vehicle or 1.5 µg/mL Lab4b CM were counted over a 7-day period. Data are presented as linear regression of percentage change in cell number carried out using GraphPad Prism 9 from three independent experiments (** $p \leq 0.01$).

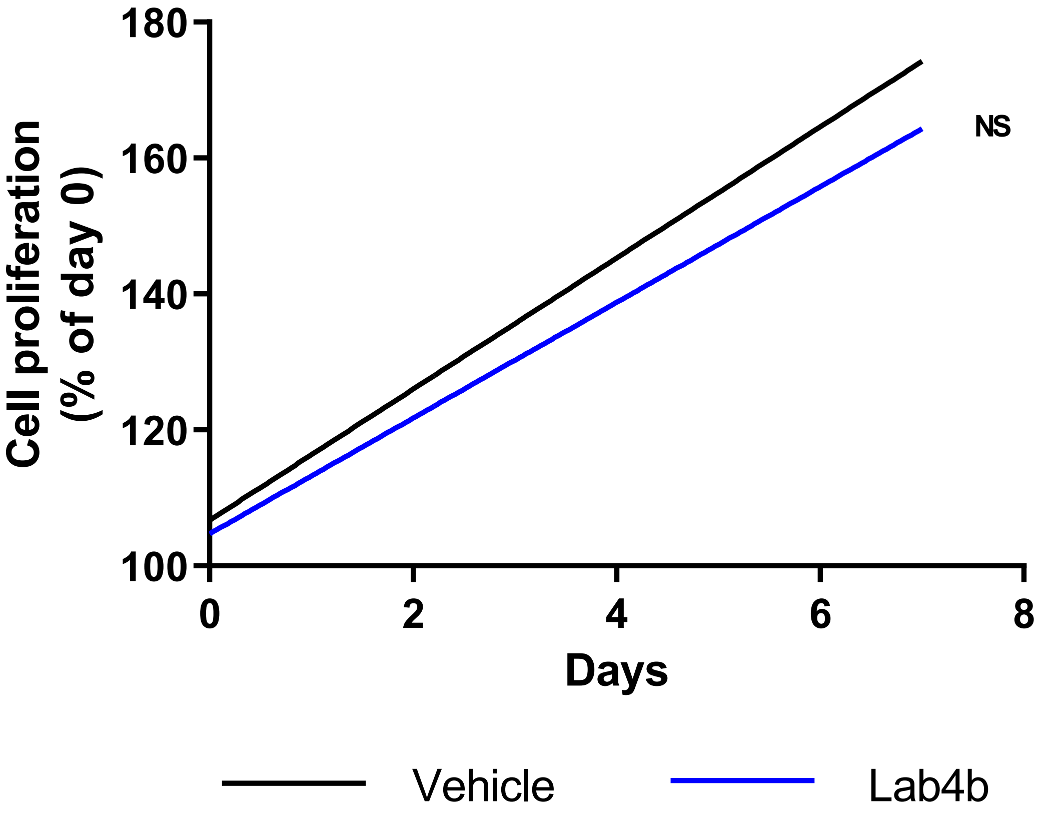

Figure S3. The effect of Lab4b CM on proliferation of HASMC over 7-day period. HASMC were treated with either vehicle or 1.5 µg/mL of Lab4b CM, and cellular proliferation was assessed by crystal violet staining over a 7-day period. Linear regression of percentage change in cell numbers from day 0 was determined from three independent experiments using GraphPad Prism 9 (NS, not significant).
